# Micro-coil embolization for transcatheter septal ablation in a hypertrophic obstructive cardiomyopathy patient and an unusual coronary anatomy: a case report

**DOI:** 10.3389/fcvm.2025.1637475

**Published:** 2025-11-14

**Authors:** Aleksandar Mandic, Gorica Vidovic, Dragana Radoicic, Ivan Ilic

**Affiliations:** 1 Department of Cardiology, Institute for Cardiovascular Diseases Dedinje, University of Belgrade, Belgrade, Serbia; 2 Department of Non-Invasive Radiological Diagnostics, Institute for Cardiovascular Diseases Dedinje, University of Belgrade, Belgrade, Serbia; 3 Faculty of Medicine, University of Belgrade, Belgrade, Serbia

**Keywords:** alcohol septal ablation, hypertrophic cardiomyopathy, micro-coil, "single" coronary artery, coronary anomaly

## Abstract

**Background:**

Transcatheter septal ablation is a minimally invasive therapeutic method for treating symptomatic obstructive hypertrophic cardiomyopathy (HCM). The procedure is an alternative to septal myectomy, and it can be achieved using different modalities. Coronary artery congenital anomalies can coexist with HCM and can add complexity to its treatment.

**Case summary:**

A 65-year-old female presented with angina, reduced exercise tolerance, palpitations, and dizziness. Echocardiography showed eccentric left ventricular hypertrophy with basal septal thickness of 20 mm and left ventricular outflow tract (LVOT) obstruction with maximum gradient of 209 mmHg. Cardiopulmonary exercise stress test showed a decreased oxygen uptake (VO2) of 13.7 ml/min/m2. Medical therapy titrated to maximum tolerated doses failed and septal reduction therapy was indicated. Prior to alcohol septal ablation (ASA), coronary angiography revealed a rare coronary artery anomaly, a single coronary artery originating from the right coronary sinus and the procedure was unsuccessful due to kinking of the over-the-wire (OTW) balloon, caused by the acute take-off angle of the septal branch. The second attempt was done using a microcatheter that was advanced to the septal branch over a hydrophilic coronary guidewire and embolization was done with two micro-coils 2mm × 2 cm. Procedural echocardiography revealed basal septal akinesia and reduced LVOT velocity from 7.24 m/s to 1.5 m/s. After six months, the patient reported a decreased frequency of chest pain and improved exercise tolerance. Echocardiography revealed the septal thickness of 15 mm, with an LVOT gradient of 24 mmHg. Follow-up CT coronary angiography confirmed a “single” coronary artery type R-III, with separate origins of LAD and Cx from the proximal RCA. Part of LAD went through the interventricular septum, forming a myocardial bridge, classified as a potentially “malignant” S subtype.

**Conclusion:**

Micro-coil embolization is a feasible alternative treatment to alcohol septal ablation in patients with obstructive HCM and anomalous origin and the course of coronary arteries.

## Introduction

Septal reduction therapy (SRT) can be a treatment of choice for patients with the obstructive form of hypertrophic cardiomyopathy (HCM). Depending on the patients' characteristics, septal reduction can be achieved by surgical septal myectomy or an interventional procedure (1). Transcatheter septal ablation is a minimally invasive therapeutic method for the treatment of obstructive HCM in selected patients who do not respond to optimal medical therapy (2). The procedure causes ischemic myocardial infarction in the territory of the septal branch, usually of the left anterior descending (LAD) coronary artery, thus creating a scar that reduces septal hypertrophy and the obstruction in the left ventricular outflow tract (LVOT). It can be done using two alternative methods, with alcohol or micro-coil implantation (2, 3).

A “single” coronary artery (SCA) is a congenital anomaly where one coronary artery arises from the coronary sinus and supplies the entire heart. The incidence of SCA in the general population ranges from 0.019% to 0.4%, and it is often discovered incidentally during coronary angiography or computed tomography coronary angiography (CTCA). Although it is typically asymptomatic, it can lead to angina and may be associated with myocardial infarction or sudden death, even in the absence of underlying atherosclerosis (4–6).

## Case presentation

**Figure 1:**
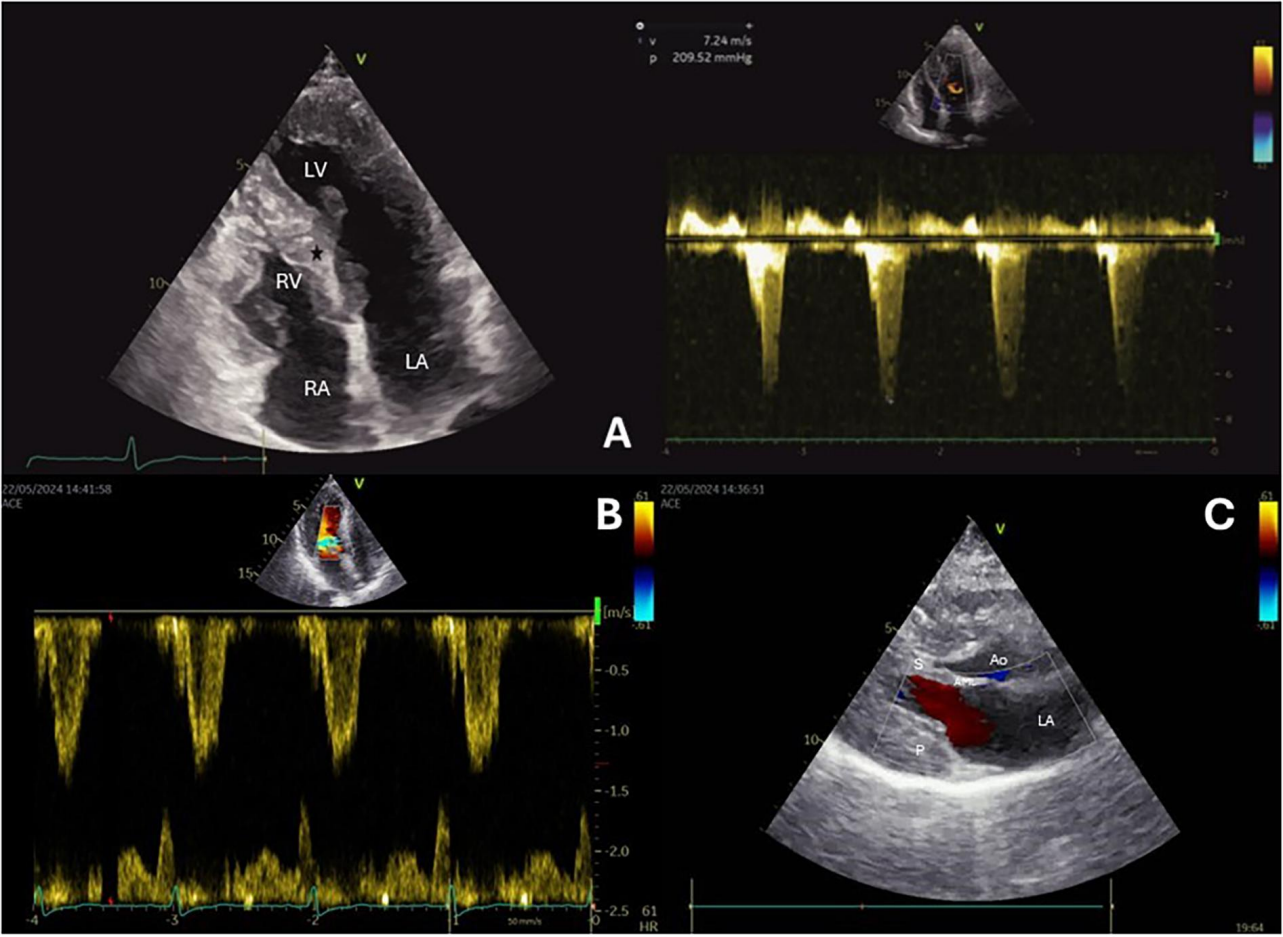
Echocardiography before the procedure **(A)** shows a hypertrophic basal segment of the interventricular septum (black star), and the continuous wave Doppler signal demonstrates increased LVOT velocity and gradient. After the procedure **(B)**, echocardiography shows reduced interventricular septal thickness, and color Doppler reveals decreased velocity in the LVOT. Color Doppler echocardiography **(C)** reveals absence of mitral regurgitation.

A 65-year-old female with a history of hypertension and asthma, presented with recurrent chest pain and severe fatigue and also experienced palpitations and dizziness. Echocardiography revealed concentric left ventricular hypertrophy with septal thickness of 20 mm, systolic anterior motion (SAM), LVOT obstruction and maximum gradient of 209 mmHg, measured during and immediately after the Valsalva maneuver (Figure 1A). Cardiac magnetic resonance (CMR) imaging confirmed echocardiography findings and transthyretin (TTR) amyloidosis and Anderson-Fabry disease were ruled out.

Cardiopulmonary exercise test (CPETx) showed mixed ventilatory disorder with decreased ventilatory efficiency during effort with VO2 max of 13.7 mL/min/m^2^ (68% of predicted value).

A 24-h Holter ECG recorded normal sinus rhythm with 425 premature ventricular complexes (PVC) in four morphological forms and one episode of non-sustained VT (4 consecutive ventricular complexes).

According to the current guidelines (6), we initiated medical treatment with a low dose of a cardio-selective beta-blocker—1.25 mg of bisoprolol. Bisoprolol was gradually uptitrated, but despite reaching the maximum tolerable dose (5 mg in the morning and 2.5 mg in the evening), the patient remained symptomatic. Alternatives such as disopyramide and cardiac myosin inhibitors (e.g., mavacamten) were not available for use in Serbia, as they have not yet been authorized by the national regulatory authority. Therefore, our Heart Team has decided to proceed with septal reduction therapy.

**Figure 2:**
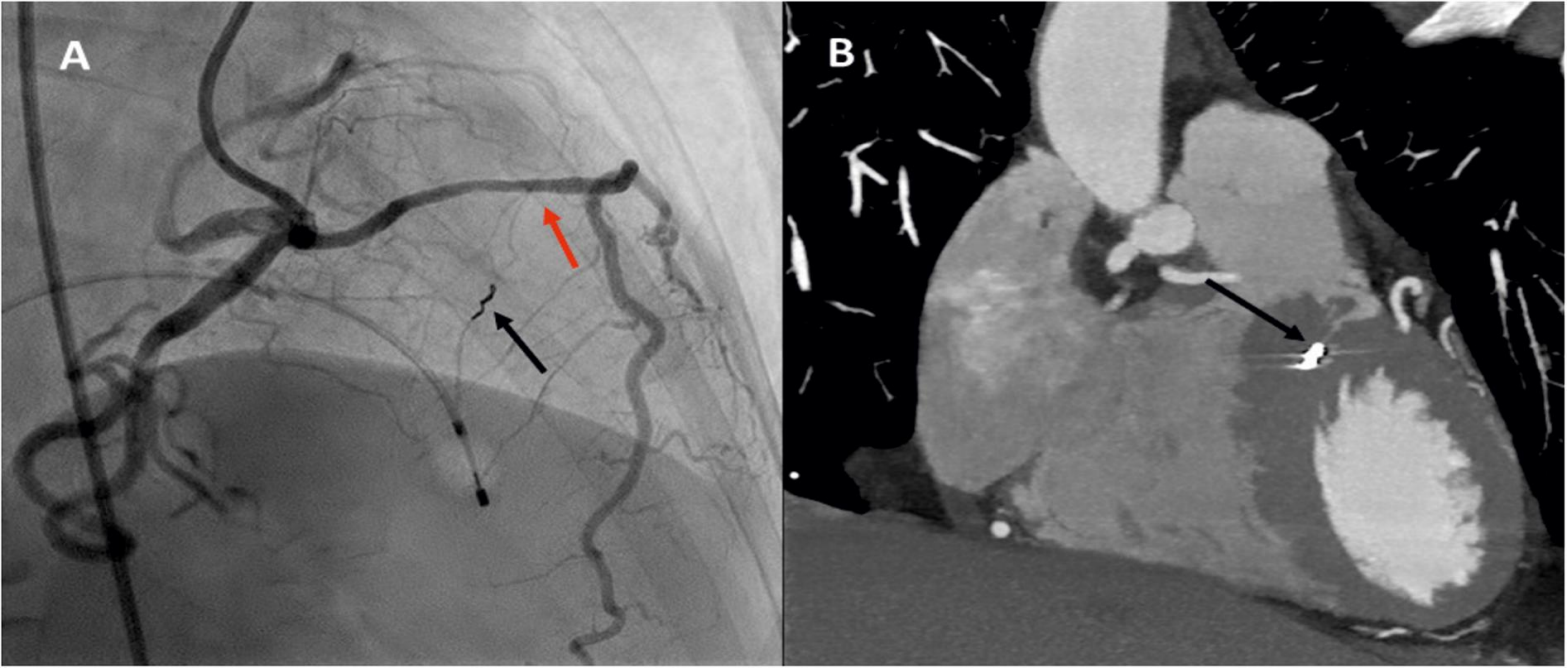
Invasive coronary angiography **(A)** and multiplanar reconstruction cardiac CT **(B)** with microcoil in septal branch acute take-off (red arrow) and microcoil (black arrow) positioned in the largest septal branch—figure 2A; micro coil in septal branch (black arrow)—figure 2B.

Initial coronary angiography revealed a single coronary artery arising from the right coronary sinus. We opted to perform alcohol septal ablation (ASA) via the right femoral approach. A pacemaker was inserted through the right femoral vein. Initial attempt to use 6 French guiding catheters failed, so Amplatz right 2.0 7 Fr guiding catheter was used, but due to the acute take-off angle, wiring of the septal branch turned out to be challenging. Several guidewires failed, and ultimately, Whisper Extra Support (Abbott Cardiovascular, Plymouth, MN, US) was positioned in the septal branch with the support of Fine Cross microcatheter (Terumo Europe, Leuven, Belgium) (Figure 2A), followed by over-the-wire (OTW) balloon Emerge 1.5 × 8 mm (Boston Scientific International, Voisins-le-Bretonneux, France). Due to an unfavorable take-off angle, kinking of the balloon shaft occurred, preventing any fluid injection. The procedure was aborted, and a new attempt was planned.

On the second attempt, same guiding catheter and coronary guidewires were used. The embolization of septal branch was successfully done with two 2mm × 2 cm Azur Cx micro coil (Terumo Europe, Leuven, Belgium). (Figures 2A,B) During the procedure, echocardiography confirmed akinesia of the basal and mid septum, with a reduction in the LVOT velocities from 7.24 to 1.5 m/s, leading to a corresponding reduction in LVOT gradient from 209 mmHg to 9 mmHg. The procedure was completed successfully (Figures 1A–C), and the patient was discharged after 3 days with telemetry monitoring.

### Follow-up and outcome

At routine follow-ups conducted one- and six-month post procedure, the patient reported fewer chest pain episodes and improved exercise tolerance. Echocardiography revealed a septal thickness of 15 mm and an LVOT gradient of 24 mmHg. Repeated CPETx showed decreased ventilatory efficiency with VO2 of 16.02 mL/min/m^2^ (82%).

**Figure 3:**
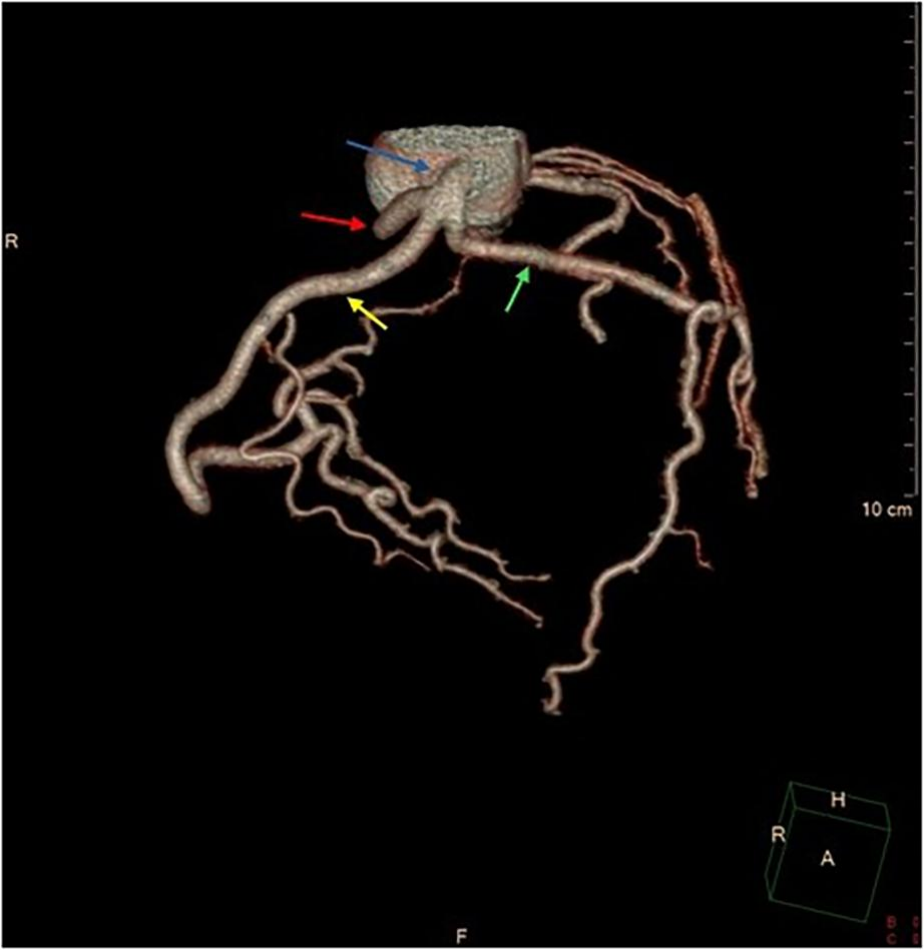
3d volume rendering cardiac CT presentation of “single” coronary artery main vessel originates from right coronary sinus (blue arrow), LAD (green arrow), LCx (red arrow) and RCA (yellow arrow).

**Figure 4:**
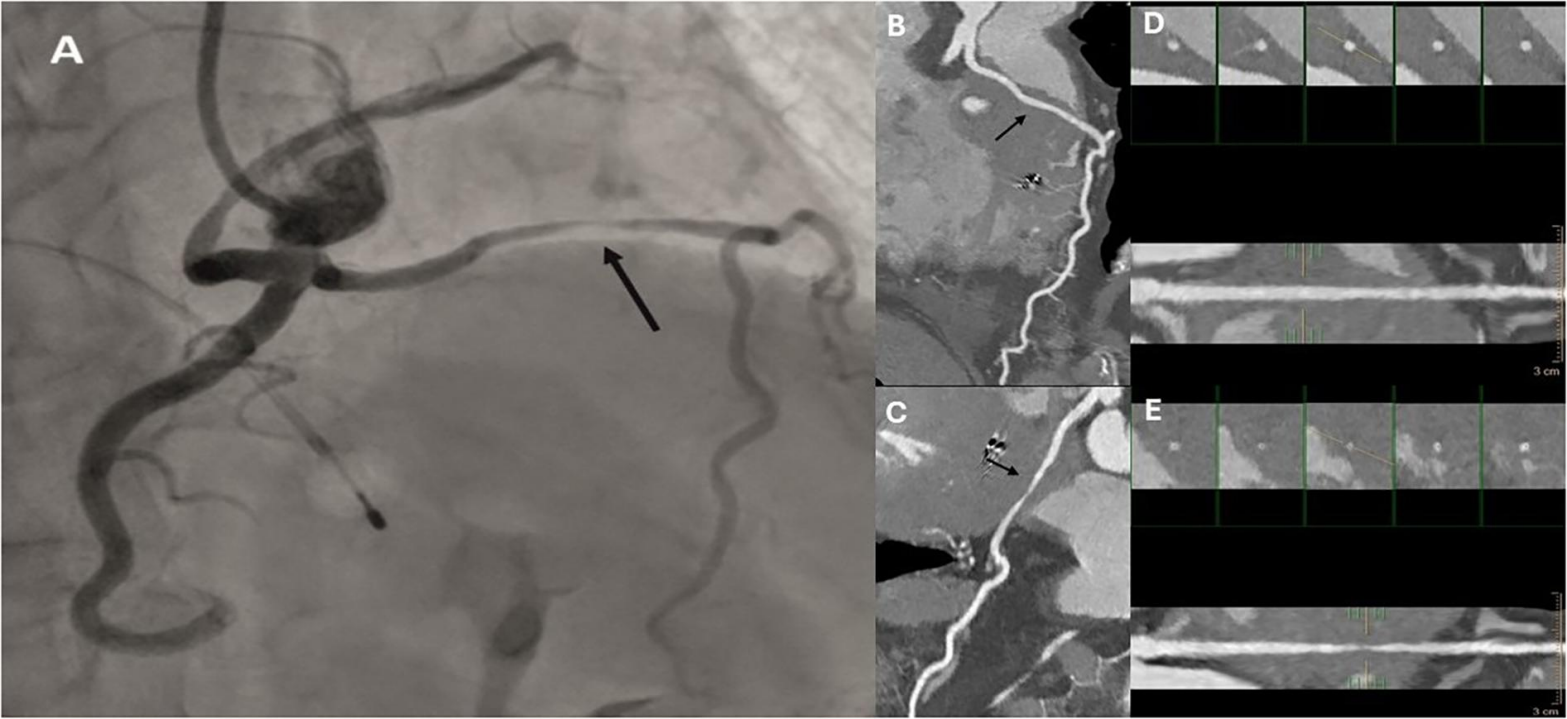
Invasive coronary angiography **(A)**, curved multiplanar reconstructions **(B,C)**, and cross-sectional vessel analysis **(D,E)** demonstrating a myocardial bridge (black arrows). Images are shown in both diastolic **(B,D)** and systolic **(C,E)** phases of the cardiac cycle, highlighting dynamic compression of the bridged coronary segment.

At six months post-procedure, CTCA demonstrated occlusion of the septal branch, with prominent metallic artifact at the site of coil deployment and absence of post-contrast opacification both proximal and distal to the coil, consistent with a lack of residual flow through the septal branch (Figure 2B). Additionally, CTCA identified a rare congenital coronary anomaly consistent with an SCA, which was classified as type R-III. In this variant, the LAD and LCx arteries originate separately from the proximal segment of the normally positioned right coronary artery (RCA), with absence of a left main coronary artery (LCA) (Figure 3, Supplementary Video S1–S2). A segment of the LAD courses through the interventricular septum, forming a myocardial bridge, which is considered a potentially “malignant” S subtype (Figures 4A–E).

## Discussion

We presented the case of septal reduction treatment with micro-coil in a symptomatic HCM patient with SCA. To the best of our knowledge, this is the first case report of such treatment.

Coronary artery anomalies (CAAs) are congenital abnormalities affecting the origin, course, or termination of the coronary arteries. They are typically classified into three categories: anomalies of origin, course, and termination (4, 7). Although most CAAs are benign and discovered incidentally, interarterial or intramural variants are associated with an increased risk of myocardial ischemia and sudden cardiac death (4, 8).

In addition to hypertrophic obstructive cardiomyopathy (HOCM), our patient had a rare congenital coronary artery anomaly characterized by an anomalous origin, classified as SCA type III-R, and a course deviation classified as type “S”. Type “S” refers to a segment of the artery passing through the interventricular septum, also known as a myocardial bridge. This is considered potentially “malignant” because the coronary artery can become compressed during exercise where increased myocardial contractility can produce intermittent occlusion, raising the risk of malignant arrhythmias, myocardial infarction and death (5, 6, 9).

Interventions in anomalous coronary vessels, such as percutaneous coronary intervention (PCI) and ASA, are technically challenging due to atypical vessel geometry, sharp ostial angles, and altered hemodynamics, which increase the risk of thrombosis, restenosis, perforation, or non-target infarction. Anomalous origin or tortuosity may compromise catheter stability and selective perfusion (4). Often, these patients present with an acute coronary syndrome or resuscitated sudden cardiac death requiring immediate coronary angiography and intervention to restore myocardial perfusion (10). Although careful pre-procedural imaging with CT angiography, MRI, or intravascular ultrasound, combined with multidisciplinary planning, is essential to define anatomy and minimize procedural risks, the complexity of CAA is sometimes appreciated only after emergency intervention (9, 10). High-risk variants may be better managed surgically if there is evidence of myocardial ischemia or malignant arrhythmia attributed to CAA (4).

The initial attempt of septal reduction with alcohol was unsuccessful due to the extreme take-off angle of the septal branch that lead to kinking of the OTW balloon, which made the procedure unattainable.

Septal reduction therapy (SRT), an invasive treatment for reducing left ventricular outflow tract obstruction (LVOTO), is recommended as the preferred approach for patients with an LVOT obstruction gradient of ≥50 mmHg and severe symptoms (NYHA class III–IV). It can be considered in patients with moderate symptoms (NYHA class II) who remain symptomatic despite optimal medical therapy, provided they have a resting or maximum provoked very high gradients or repetitive bouts of atrial fibrillation (11, 12, 13).

Ventricular septal myectomy (Morrow procedure) remains mainstay surgical intervention for treating obstructive HCM. However, transcatheter septal ablation has become an acceptable alternative (12–14). The large retrospective studies showed that in-hospital mortality following surgical myectomy may be significant, averaging 4%, with an inverse relationship between procedural volume and mortality—meaning that centers with higher volumes had lower mortality rates. In contrast, mortality after ASA was similar across all volume tertiles, suggesting consistent outcomes regardless of procedure volume (15, 16). These findings influenced the decision-making process of our Heart team, particularly in light of the limited experience with septal myectomy at our Institute. It can be achieved using two main techniques: (1) ASA or (2) non-ASA methods, such as embolization of the septal branch with coils, polyvinyl alcohol foam particles, and cyanoacrylate (3, 11, 17). Alternatives, such as direct endocavitary or intramuscular ablation (using radiofrequency or cryotherapy), are also available, but these techniques are generally less effective than other SRT methods (11).

Despite relatively simple interventional technique, compared to surgical treatment, ASA has limitation regarding the penetration in heavily thickened septa, concomitant mitral valve disease and need for pacemaker implantation and repeated interventions (18, 19). However, coil embolization offers some advantages, such as a more localized area of myocardial infarction, restricted primarily to the basal interventricular septum (IVS), less damage to the conductive tissue, preventing unintended spread of ethanol to other myocardial areas, and a lower risk of complete heart block (CHB) (20). While there is limited data, some studies have shown that coil embolization is non-inferior to ASA (3, 17). These studies report significant symptom improvement in 90% of cases and a reduction in the LVOT gradient in 75% of cases, though only a few have included long-term follow-up (3). On the other hand, the downside of this technique could be the absence of immediate effects of the intervention, in terms of LVOT gradient reduction, which sometimes requires repeated invasive procedures in order to obtain the optimal result.

Although surgical treatment of myocardial bridging is a therapeutic option, there is currently insufficient evidence to unequivocally support this treatment (21). Furthermore, surgical management of coexisting HOCM and coronary artery anomalies would be technically highly demanding. The patient was evaluated using SCD risk score (11) and the risk was estimated to be 1.04% and ICD was not deemed necessary. Repeated CPETx and echocardiography would be used for further monitoring the patient's risk of adverse cardiovascular events during follow up.

The complex coronary anatomy required modification of interventional techniques in terms of using larger diameter guiding catheters, hydrophilic wires and microcatheters in order to reach difficult septal anatomy. Although alcohol septal ablation in general is not considered a complex intervention, in case of coronary artery anomalies it can become challenging (11, 14).

## Conclusion

Micro-coil embolization is a feasible alternative treatment to alcohol septal ablation in patients with obstructive HCM and anomalous origin and the course of coronary arteries. This technique should be the integral part of the armamentarium in an expert center treating patients with obstructive HCM.

## Data Availability

The original contributions presented in the study are included in the article/Supplementary Material, further inquiries can be directed to the corresponding author.

## Supplementary material

Supplementary Video S1 Three-dimensional (3D) volume-rendered reconstruction demonstrating the anatomical origin of a single coronary artery from the sinus of Valsalva and the subsequent course of its major branches. The dataset was generated from high-resolution CCTA. The rendered model is continuously rotated 360 degrees around the axial plane.

Supplementary Video S2 Three-dimensional (3D) volume-rendered reconstruction depicting the ascending aorta, the anatomical origin of a single coronary artery from the aortic bulb (sinus of Valsalva), and the course of its major branches. The dataset was generated from high-resolution CCTA. The rendered model is continuously rotated 360 degrees around the axial plane.
